# The Management of Cow Shelters (Gaushalas) in India, Including the Attitudes of Shelter Managers to Cow Welfare

**DOI:** 10.3390/ani10020211

**Published:** 2020-01-28

**Authors:** Arvind Sharma, Catherine Schuetze, Clive J.C. Phillips

**Affiliations:** 1 Centre for Animal Welfare and Ethics, School of Veterinary Science, The University of Queensland, Gatton Campus 4343, Australia; c.phillips@uq.edu.au; 2 Faculty of Arts and Social Sciences, The University of Sydney, New South Wales 2006, Australia; vajracat@gmail.com

**Keywords:** shelters, cows, managers, survey, attitudes, welfare, India

## Abstract

**Simple Summary:**

Sheltering of old, unproductive and abandoned cows in traditional cow shelters (gaushalas) is an ancient practice in India. Cows are venerated as mother goddesses by the Hindu majority population of the country and their slaughter is illegal in most states. Shelters are funded by the public, businesses, including corporate philanthropy, charitable societies, temple trusts and government. The manager of the shelter provides an interface between visitors, workers and cattle and is best able to understand the challenges of running shelters. The objective of this study was to collect and analyze information about the routine operations of the shelters and elicit managers’ attitudes towards cows and cow welfare. We visited 54 shelters, which admitted cattle all year, vaccinated them against endemic diseases and dewormed them. Limited biosecurity measures and erratic waste disposal raise concerns about public health. All the managers felt that the welfare of cows in their respective shelters was important and should be improved, but they were less certain that their knowledge of animal welfare was adequate. There was more recognition of local community support than government support and both were acknowledged to be more moral than financial support. Engagement and training of shelter managers as vital stakeholders in welfare improvement processes will enhance the sustainability of these traditional institutions.

**Abstract:**

Gaushala management is a specialized profession requiring particular skills relating to the management of cow shelters or gaushalas, which are traditional and ancient Indian institutions that shelter old, unproductive and abandoned cows, The 1800 registered cow shelters in India have managers who are important stakeholders in the management of cows in these unique institutions. It is important to survey the routine management of these shelters and attitudes of the managers towards cow welfare to identify the constraints and welfare issues. We visited 54 shelters in six states of India for a face-to-face structured interview of the managers. Quantitative data collection included questions on demographics, routine management operations, protocols followed in the shelters and attitudes of the managers towards cow welfare. All shelters except one were managed by males, half of them were in the age range of 45–65 years, were university graduates or post-graduates, with 5–15 years shelter management experience, and with the majority having lived in rural areas for most of their lives. Each shelter housed a median of 232 cattle were housed, out of which 13 were lactating cows. The majority of managers vaccinated their animals against endemic diseases like foot and mouth disease, haemorrhagic septicaemia and black quarter (gangraena emphysematosa) and administered endo-and ectoparasiticidal treatments, however, hardly any screened the cattle for brucellosis and tuberculosis. Only 17% of the shelters had in house veterinarians and most cows died of old age, with an annual mortality rate of 14%. The majority of the shelters allowed the cows to reproduce. Access to pasture was available in only 41% of the shelters, while most allowed some access to yards. Most (57%) had limited biosecurity measures, but 82% of the shelters disposed of the carcasses by deep burial on their own premises or through the municipality, with 18% disposing of them in open spaces or nearby creeks. About one half of the shelters maintained records of the protocols followed routinely. Charitable societies ran half of the shelters, mostly through public donations, with accounts audited regularly. Most managers thought that shelter cows’ welfare was important and that they should attempt to improve it. They were less in agreement that their knowledge of animal welfare was adequate. Local support, more moral than financial, was recognized more than government support. Managers perceived cow welfare as important from a religious perspective, citing the mother god and caring for abandoned animals as frequent themes in their definition of cow welfare. Caring for animals, mother and goddess were key elements in managers’ perception of animal welfare. The recommendations arising from this survey include that the shelter managers should be involved in the decision-making process for the welfare of cows in shelters, which is vital for the sustainability of these unique institutions. Welfare could be improved by strict compliance with biosecurity measures and disease surveillance protocols, avoidance of unrestricted reproduction in cows and separation of males and females.

## 1. Introduction

In India cows in their late lactation, with reduced production and competing with other cows for the costly feed, are often abandoned to the streets. In urban areas, they then forage on garbage dumps, potentially consuming plastics and wires, as well as potentially suffering fatal traffic injuries [1]. Abandoning of cows in streets is contentious as these cows are often injured, even causing human mortality, and potentially causing a public health risks to humans and animals [2,3]. According to the Indian government, stray animals caused 1604 road accidents in 2016, leading to 629 human deaths [4]. Stray cows in the roads and streets have specifically been implicated as the causes of these road accidents [5]. In the villages, crop-raiding by abandoned cows has led to human–animal conflict, with farmers sometimes having to abandon cropping and cows beaten and chased away. In this scenario, gaushalas are the only alternatives to shelter these stray cows, as a religious ban on cow slaughter is increasing their numbers every year. 

The sheltering of old, abandoned, unproductive, infertile and infirm cows in shelters, referred to as “Gaushalas” is a traditional practice in India. The exact origin of these shelters is not known but documentary evidence of their existence is available from the 3rd to 4th century BCE [6]. Over time they diversified, based on their religious affiliations and ownership [7]. Cows are worshipped as a mother goddess by many Hindus, who constitute the majority population. Cow slaughter is illegal in most Indian states [8,9]. Both the Muslim invasion and the later European colonization created socio-political conditions linking the cow with symbols of purity and Hindu identity. More recently, political parties strengthened the cow protection and cow sheltering movement [10,11,12]. Mahatma Gandhi emphasized the role of shelters in the economic growth of India rather than their religious role, by advocating the dairying and breeding of shelter cows based on the scientific principles [13]. In the early independence years, the role of gaushalas changed from sacred cow sanctuaries to potential breeding and dairying centers for high yielding cows, with active financial support from the government [14].

India is the largest producer of milk and has the largest number of dairy cows in the world (58.5 million), as well as the largest cattle population (190.9 million). The last livestock census (2012) reported 5.2 million stray cattle [15]. In a government survey conducted in 1956 there were 1020 gaushalas in 21 states of India [16], which has grown to the current 1837 registered gaushalas, according to the Animal Welfare Board of India (AWBI), the statutory body under the Government of India’s Prevention of Cruelty to Animals Act 1960 (PCA, 1960). However, there are reports that the total number, including unregistered gaushalas, is approximately 5000 [17,18].

Managers are employed by the trustees, charitable societies, temple trusts, municipalities or government, according to who owns the shelter. A two thousand-year-old Hindu text, the ‘Arthashastra’, describes the administration of gaushalas, including a position of ‘Godyaksa’ (Superintendent of Cows) [6]. Nowadays managers provide an interface with visitors, who come to donate, worship, feed or just see the cows. Managers have multiple roles, as cashiers, cattle and worker superintendents, and receptionists. Despite this, their attitudes towards cow welfare and gaushalas have never been studied. Studies investigating attitudes towards animal welfare issues are common in developed countries [19,20,21], including aspects of dairy farm management [22,23,24], and even dairy farms in India [25,26,27].

The paucity of studies on gaushala management is evident [6,28,29], even though there are qualitative studies critical of the management of cow shelters in a philosophical context [9,12]. There is a lacuna in literature on the quantitative assessment of the routine management of cow shelters in the contemporary context, when the sheltering of cattle has gained importance in the wake of an increasing problem of street cows and the impetus for strengthening the shelters from the Indian government.

Therefore, a survey was designed to collect and analyze information about the routine animal husbandry operations and practices of shelters and to elicit the attitudes of managers of the gaushalas to cows and their welfare. Information about the routine working of the gaushalas, husbandry practices followed, demographics of the sheltered animals, preventative health and biosecurity measures undertaken, income and expenditure of the gaushalas, constraints and visitor profile are important to objectively assess the welfare of the cows in these shelters. The opinions and attitudes of these managers towards cows and cow welfare is also important to provide feedback to the stakeholders—shelter owners, donors, trustees and the government. This feedback can help in initiating training programs and selecting appropriate candidates for recruitment as shelter managers.

## 2. Materials and Methods

**Figure 1:**
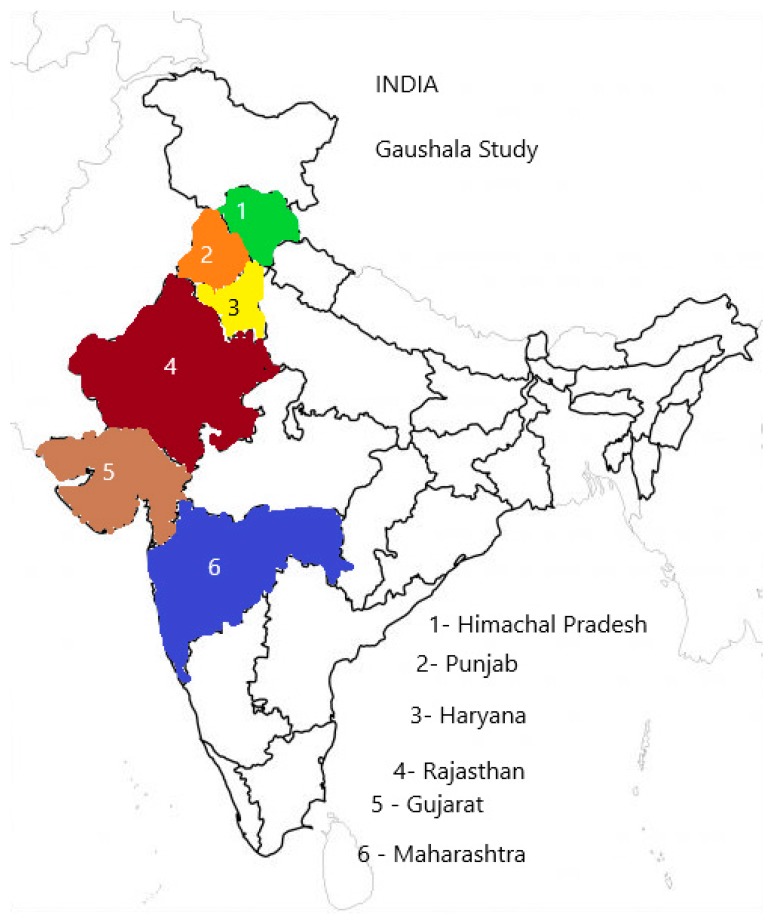
Schematic map of India depicting states covered under the gaushala study.

Human ethics approval for this study was provided by the University of Queensland’s Human Ethics Committee (approval number 2016001243). Interviews were conducted with shelter managers between November 2016 and July 2017, as a part of a welfare assessment of cows in shelters in six states of India (Gujarat, Maharashtra, Rajasthan, Punjab, Haryana and Himachal Pradesh) [30]. These states were selected on the basis of having the largest concentration of shelters in India and a tradition of sheltering cows (Gujarat, Rajasthan, Maharashtra, Punjab and Haryana) and one state (Himachal Pradesh), which was actively establishing cow shelters to tackle the stray cattle problem (Figure 1). As there is no list of all shelters in India, a list of shelters supported by the AWBI was used in the selection process, which was to an extent random but had to consider the logistics for visiting them. Local veterinarians and shelter staff helped in locating the shelters and introducing the interviewer to the managers. The local veterinarians and shelter staff had no role in influencing the managers’ responses to our questionnaire or in any other way affecting the assessment of the shelters. Each shelter manager of the 54 cow shelters assessed was interviewed for approximately 35 min, before assessing the animals and resources present for the objective assessment of the overall welfare of cows in shelters. The sample size of shelters (n = 54) was determined using a power calculation [31] which determined that 50 shelters would adequately represent the number of shelters in major Indian states having shelters. The study was designed to detect an odds ratio of 4 with a power of 0.8 and α = 0.05. The prerequisite for shelter selection was that they should be sheltering at least 30 cattle and should not be selling more than 20 litres of milk per day. A good geographical distribution of the shelters in each state was ensured for sampling in the study along with a mixture of good or bad shelters. Shelters were selected on the basis of recommendations of the AWBI, veterinarians working in the state animal husbandry departments and through a snowballing technique.

### Questionnaire Design

Interviews with the shelter managers in Hindi were conducted using a questionnaire with multiple-choice, semi-closed and open-ended questions, to collect socio-demographic data, data about shelter management and husbandry practices and attitudinal data of the managers to cows and cow welfare (Appendix A). The first section had three screening questions about whether the shelter housed at least 30 animals, whether infertile, abandoned, rescued, stray, old and infirm cows were being sheltered, whether the shelter had any religious connection and age of the shelter. The second section on demographics asked their gender, usual place of residence, age, religion and religiosity and education level. They were then asked to describe their job in the shelter, their level of understanding and knowledge about cow shelters, source of gaining this knowledge, any animal welfare activity outside of the shelter, and the length of time they had spent working in that shelter. The third section addressed cattle numbers and cattle management: the number of lactating cows, mean milk yields, the proportion of horned cattle, the number of other cattle (bulls, bullocks, non-lactating cows and heifers, males and female calves, less than 6 months of age), the fate of calves born in the shelter (sold, donated or reared); vaccination and deworming practices, including frequency of use and for which pathogens; veterinarian involvement (in house or visiting; frequency of visits), number of male and female workers and the length of time they had worked there, whether there was induction training, whether the manager kept records, sold livestock products and ran a biogas plant at the shelter.

The fourth section asked about the status of the shelter (public or private trust, government, charitable society, board of directors, municipality, individual or any other), the source of funding, annual income and expenditure, including whether audited, affiliation with the AWBI. The fifth section addressed husbandry: mortality and its major causes, whether colostrum was fed to calves, whether cows and calves were separated after birth, the cattle feeding regime, including whether visitors fed the cows, the time spent by the cattle outdoors in the yard or at pasture, whether the cows bred or not, and if they did the purpose of the breeding; whether there were any animal enrichment and/or biosecurity measures (the latter particularly during the introduction of new animals, disposal of carcasses, and isolation of diseased animals), the disposal of cow excreta, the maintenance of cows in segregated groups; use of loading/unloading ramps; whether animal experimentation was allowed; natural disasters plans; volunteering by the public, and any public relation or outreach activity done by the shelter.

Finally managers responded to attitude questions on a Likert scale (1, strongly disagree to 5, strongly agree): the welfare of this gaushala’s cows is satisfactory and important to me; my knowledge of animal welfare is adequate; the feed the cows receive is adequate; I am willing to adopt measures that will improve the welfare of the cows, if provided to me; the local community and government financially and morally support the gaushala; I intend to make improvements to the welfare of the cows under my care; in the past I have tried to make improvements to the welfare of the cows in my care; the staff at this gaushala have a close relationship with the cows. Finally, an open-ended question was asked: what you understand by the term ‘welfare of cows’?

## 3. Statistical Analysis

Data was screened for errors, and analysis completed with statistical software [Minitab 17 Statistical Software (2010). State College, PA: Minitab, Inc. (www.minitab.com)]. Descriptive statistical analysis was performed on the questionnaire results and respondent demographics, complimentary data, and responses to attitude questions (refer Supplementary Materials) expressed as numbers and percentages.

The association of the dependent variables, income of the shelter and mortality rate of cow shelter with various categorical and continuous independent variables was explored using a general linear model (GLM). Logistic regression analyses (either binary, nominal or ordinal, as appropriate to the response structure) were used to analyze the significance of relationships between type of administration, affiliation with AWBI and financial support of the government (which had Likert scale response), the income of the shelter, mortality rate, disease outbreaks in the last five years, biosecurity measures, breeding of cows in shelters, time cows spent outdoors, training of workers, frequency of veterinarian visits, frequency of deworming, ectoparasiticidal treatments and vaccination, numbers lactating cows in the shelters, total milk yield of the shelters and the total number of animals in the shelters. Cross tabulations between dependent variables and independent variables were also inspected, ensuring that all individual expected counts were ≥1. An iterative reweighted least squares algorithm with a logit link function was used in the models. All models achieved convergence. All probability values were considered significant at *p* < 0.05.

The type of administration of the shelter (whether managed by a public trust, private trust, government or a charitable society), Affiliation with the Animal Welfare Board of India (AWBI) and Income of the shelter were used as outcome variables against animal health and welfare based variables: mortality rate, vaccination status, vaccination frequency, status and frequency of deworming and ectoparasiticidal treatment, total number of animals in the shelter, milk yield of cows in the shelter, number of dairy cows in the shelter, frequency of veterinarian visits to the shelter, training of workers, biosecurity measures for new cattle admitted, time spent by the cows outdoors and disease outbreaks in five years. According to the nature of outcome variable (continuous, binary or ordinal) GLM, ordinal or nominal regression models were used to explore associations between these variables.

We used a one-way ANOVA to determine whether any significant differences in the responses to the twelve attitude questions existed. Each attitude question was taken as a response and the other 11 questions were used as factors with the possible answers to each question as levels of the factor variable (1, strongly disagree to 5, strongly agree). The level of significance was fixed at 5%. Tukey’s method was used to compare the means for each pair of factor levels to control the rate of type 1 error. Chi-square test for association was used to test for any differences in the disposal of male and female calves.

Thematic analysis of the open-ended question about what the gaushala manager understood by the term ‘welfare of cows’ was conducted by a single thematic coder, using NVivo Pro 12 software (NVivo qualitative data analysis software; QSR International Pty Ltd. Version 12, 2018, https://www.qsrinternational.com/nvivo/nvivo-products/nvivo-12-plus). This extracted the main trends from the word frequency and word cloud functions. Conjunctives (such as ‘and’) and words which were irrelevant to the study theme (such as ‘a’ or ‘it’) were manually excluded from the output and the analysis repeated.

## 4. Results

### 4.1. Respondent Demographics

All 54 shelter managers completed the questionnaire, a 100% response rate. There was only one female shelter manager. The majority of the managers had lived most of their lives in villages (63%), some in urban areas (28%), country towns (7%) and suburban areas (2%). Most were aged 46–55 years (26%), or over 65 years (22%), with fewer 36–45 years (18%), 56–65 years (17%), 26–35 years (15%) and 18–25 years (2%). Of the managers, 28%were university graduates, 24% were post-graduates, 21% ended their education after passing grade 12 and 9% at grade 10, 13% were diploma holders, and 5% were either below grade 10 pass or had no formal education.

Almost all of the managers were Hindus (96.3%), with many considering themselves very (55.5%) or moderately (43%) religious. Nearly all (94%) considered their job as being team leaders supervising staff working directly with the cows; only 6% indicated that they worked directly with the cows. The majority of the managers (67%) believed themselves to have a good knowledge and understanding of cow shelters, 18% considered themselves to be experts, 13% considered that they had some knowledge and 2% little knowledge. A majority (81%) indicated that their knowledge of cow welfare came from hands-on experience of working on farms, 7% had formal qualifications on welfare, and 3% from newspapers, periodicals, television programs and the internet. Although most (59%) were not involved with any animal welfare organisations, some (61%) were involved in other animal welfare activities: animal activism, humane education or feeding stray animals. Only 30% were involved with professions unrelated to animal welfare before joining the shelters, 33% had a long experience of similar work in animal welfare, more than 15 years, followed by 21% between 5–9 years, 17% between 10–15 years, 13% between 2–3 years, 11% between 3–5 years and only 5% being there for less than a year. Of the managers, 28% had spent 10–15 years working at their current shelter, followed by 19% 3–5 years, 17% 5–9 years, 17% >15 years, 13% 2–3 years and 7% < one year.

### 4.2. Establishment of the Shelters and Their Financial Performance

Half of the managers reported the shelter’s religious connection to Hinduism (27 shelters), 11% to Jainism, 9% to Jainism and Hinduism, 8% to others (Sikhism and Islam), and 22% had no religious connection. The oldest shelter in our study was established in the year 1766 according to the records available to the shelter managers, five shelters were established in the 19th century, five in the first half of the 20th century and the rest were established in the second half of the 20th century and in the 21st century. Almost half of the shelters (48%) visited in this study were administered through charitable societies, 33% by public trusts, 13% by private trusts and the rest (6%) by government, municipalities or temple trusts. Philanthropy by the public, business houses, trusts and funding by the state governments were the principal sources of funding for the shelters. Only 46% of the shelters were affiliated to the AWBI. Regular auditing of the shelter funds was done in 96% of the shelters.

Out of the 54 shelter managers interviewed in the study, 50 provided the estimated income and expenditure of their shelters. The median annual expenditure of the shelters was 3,525,000 Indian rupees (approximately US$ 50,000). The median annual income was 125,000 rupees (approximately US$ 1800). The maximum annual expenditure being incurred by a shelter was 150,000,000 Indian rupees (approximately US$ 2,000,000). In addition, five of the shelters reported no incomes and the maximum annual income reported was 12,444,000 Indian rupees (approximately US$ 174,000).

Income was provided by sales of milk, manure, urine and hides. Milk was sold in only 37% of the shelters, and most of the milk produced was distributed free of cost to the workers by the gaushala managers. Dung was sold as manure in 54% of the shelters. Partial disposal of dung by shelters was done in the form of donation of manure free of charge to the local farmers (37%), sale as manure alone (37%) and sale as vermicompost and manure (17%). Biogas as an alternative dung-generated fuel was produced in only 19% of the shelters. In 9% of the shelters dung was not disposed of but left lying as a mound within the shelter premises. In the case of urine, 76% of the shelters just let it drain off without proper sewerage facilities to treat the slurry, and 24% of the shelters collected urine to use as a biopesticide or in traditional medicine. Hides of dead animals were sold in 11% of the shelters.

Recording of milk yield in the shelters was done only in half of the shelters. Calving and mortality records were maintained in 63% and 81% of the shelters, respectively. Health records were maintained in 80% of the shelters. An inventory of veterinary drugs was maintained in 76% of the shelters. Feed records were maintained in 91% of the shelters, while 76% of the shelters maintained records of any sales.

### 4.3. Cattle, Worker and Visitor Demographics

The median number of animals housed in the shelters was 232. The median number of cows, heifers, bulls, bullocks, female and male calves were 137, 48, 12, 9, 11 and 15, respectively. The median number of lactating cows in the shelters was 13, with a median milk yield of 12 l/d/ shelter. Nearly all (90%) cattle were horned. In each shelter the calves were usually reared there (mean/shelter/year, n = 64, 59% of total calves), some donated to villagers if requested (n = 31, 29%) and a small proportion sold (n = 13, 12%), with no significant difference between males and females (Chi Square = 0.98, *p* = 0.61).

The median number of male workers was six and females two, with 32% of the shelters having no female worker. The maximum number of male and female workers in a shelter was 300 and 110, respectively. Induction training of the workers was performed in 65% of the shelters. Anecdotally, we were told that females more often worked in those shelters that provided worker accommodation within the shelter premises.

Regular volunteering in the shelters by the local public was reported in 30% of the shelters, occasional volunteering in 26% of the shelters and the absence of volunteering in 44%. In order to have an outreach to the public, 72% of the shelters organized activities such as the celebration of cow specific holy festivals (like *‘gau ashtami’, ‘govardhan pooja’*), recitation of religious scriptures by saints, open days and community feasts, according to their financial capacities.

All shelters allowed visits for a variety of purposes: exclusively for religious reasons was reported by 9% of the managers, 39% for seeing or feeding the cows and 52% for all the above reasons. Most of the shelters (98%) did not allow anyone to conduct experiments on their animals. Nearly all shelters (96%) allowed visitors to feed the cattle, and 87% of the shelters monitored it. Most of the shelters allowed feeding of homemade food to cows by the visitors after proper monitoring of the contents of the food. However, on special occasions, there were more visitors offering food to the cows.

### 4.4. Health Management, Breeding, Housing and Disaster Management

Almost all the shelters (96%) vaccinated their cattle against foot and mouth disease (FMD), haemorrhagic septicaemia (HS) and black quarter disease (BQ) in 85% of the shelters and FMD and HS only in 11%. There was only one shelter that vaccinated against brucellosis along with the other diseases, and one shelter did not vaccinate their animals at all. Most of the shelters (81%) vaccinated the cattle twice a year and 15% thrice a year. Endoparasiticidal treatment was given biannually in 35% of the shelters, three times a year in 17%, four times a year in 30%, once a year in 5% and three shelters never gave it. Regular schedules of endo and ectoparasiticidal treatment were used by 7% and 50% of shelters, respectively. In addition, 21% of the shelters treated four times a year, 11% twice a year, 5% three times a year, 3% once a year and 7% of the shelters never treated with either parasiticidal treatment.

Only 17% of the shelters employed in-house veterinarians but a further 26% had veterinarians on call. Some 13% of the shelters had daily veterinarian visits, 13% weekly, 13% fortnightly and 5% monthly visits. The median mortality rate of the cattle in shelters was 30 animals/year or 13.8%. Old age was ranked as the main cause of mortality (53%), followed by animals brought in in a moribund state (28%), disease (8.5%), chronic debility (5.5%), other causes (3%, such as fatal injuries due to fights within herd mates, impaction of the gastrointestinal tract with plastics) and malnutrition/ fodder shortage (2%).

Biosecurity measures in the shelters in the form of separate sheds, were followed in 57% of the shelters during the introduction of new animals, isolation wards for separating and treating sick cows (72%); disposal of carcasses took place by deep burial within the shelter premises in 43% of the shelters, whereas 39% shelters allowed the municipalities to dispose of the carcasses. However, 18% of the shelters left the carcasses in the open or just threw them in a nearby creek or ravine. Disease outbreaks in the last five years, predominantly FMD, were reported by 43% of the shelters.

The majority of the shelters (91%) allowed the cows to reproduce; 44% of which was mating by bulls housed with the cows and 44% was planned, with cows taken to bulls when oestrus was observed. The purposes of breeding was usually (56%) for indigenous breed conservation, breed improvement and increased productivity; with 44% allowing it without any purpose. Colostrum was fed to all calves born in the shelters and 94% of the shelters fed it immediately after the birth. Calves were kept with their mothers in most (57%) shelters. In 68% of shelters the cows were segregated into different sheds according to their age and length of stay. Access to pastures was available only in 41% of the shelters, whereas 81% had access to yards. Approximately the same proportion of shelters sent their cows outdoors to the yards for less than (46%) and more than (44%) 6 h. Cows were not allowed outdoors in 9% of the shelters, mostly due to the absence of yards and pastures. Loading and unloading ramps for the cows were available in 57% of the shelters.

Most shelter managers (76%) expressed ignorance about any disaster management plans for their shelters, and 74% believed that their shelter was not located in a disaster-prone area (areas prone to flooding, avalanches, landslides, and bushfires). Animal enrichment measures were employed in 52% of the shelters but were mostly restricted to the provision of playing devotional music.

### 4.5. Association of Shelter Administration, Affiliation, Income and Financial Support of Government with Various Health and Welfare Parameters

No significant association was observed between the income of the shelters with other independent variables using a General Linear Model, though there was a trend towards shelters affiliated to the AWBI having more income (*p* = 0.07). There was a significant positive association between the mortality rates with total milk yield/day (SE of coefficient = 0.001, F = 10.37, *p* = 0.004) and presence of an in-house veterinarian (SE of coefficient = 166, F = 4.86, *p* = 0.002). The r^2^ (adjusted) for the model was 61%.

There was a significant association between the type of administration of the shelters (government, public trust, private trust or a charitable society) and the presence of biosecurity measures for newly admitted animals (OR = 18.94, 95% CI 2.73–131.22, *p*-value 0.003). Shelters run by charitable societies were less likely (10/26) to use biosecurity measures for newly admitted animals than the public trust run shelters (14/18). Acknowledgement by the managers of financial support of the government to the shelters was associated with frequency of vaccination (OR = 10.23, 95% CI 1.34–78.15, *p* = 0.02). Those shelters that disagreed that government provided financial support were relatively more likely to vaccinate their cattle twice a year (5/13) than those who agreed that government provided financial support (3/21).

### 4.6. Attitude of Managers to Cow Welfare and Support for the Shelter

**Figure 2:**
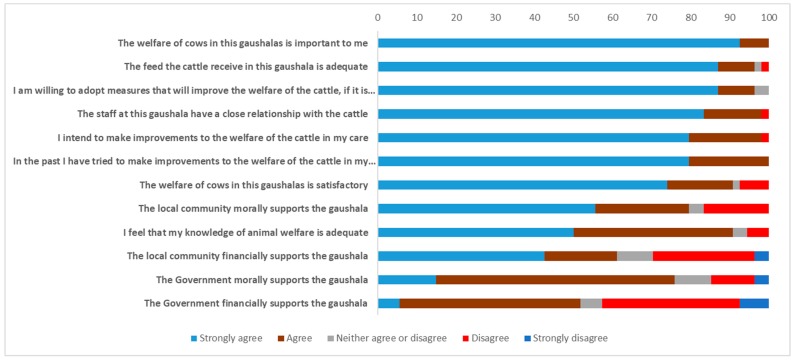
Perceived beliefs and attitudes expressed by 54 gaushala managers.

Managers’ attitudes are presented as bar charts (Figure 2), with comparison between mean responses presented in Appendix B. Most managers agreed that welfare was important to them (Figure 2), that they were willing to adopt measures to improve welfare, that feed was adequate and that they had made or intended to make welfare improvements. There was less agreement that their knowledge of animal welfare was adequate and that the local community morally supported the shelter. There was only marginal agreement that the local community morally and financially supported the shelter and that the government morally supported the shelter. There was no clear agreement that government financially supported the shelter.

### 4.7. Qualitative Assessment

**Figure 3:**
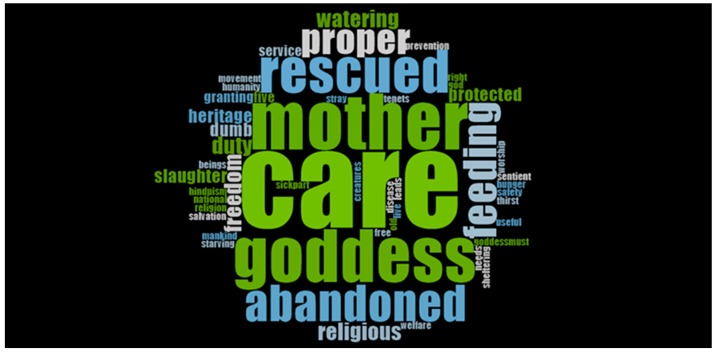
Word Cloud for the question ‘What do you understand by the term ‘welfare of cows’?

All the gaushala managers answered the open-ended question: What do you understand by the term ‘welfare of cows’? We developed 50 word frequencies from the responses (Table 1). Words that were found more than eight times were as follows: care (n = 27), mother (16), goddess (16), rescued (12), abandoned (10), feeding (9) and proper (8). The word cloud (Figure 3) emphasizes the interrelated concepts: mother, care, goddess, abandoned and rescue.

## 5. Discussion

Gaushala management in the contemporary context is challenging and complex due to the regular influx of cattle of different age groups and varied health and body condition. The managers’ performance is under the constant scrutiny by the trustees/board of directors and the public, due to the religious status of the cow in Indian society and the high expectations of the shelters to provide good welfare to the cows sheltered in them. This study is the first to report on routine gaushala management and husbandry practices across the North Western, Northern and Western parts of India, which have the highest concentration of gaushalas in the country. Overall, several positive and negative aspects of welfare and management were identified that deserve the attention of all stakeholders to improve these traditional institutions and increase their sustainability.

### 5.1. Human and Cattle Demographics

Mostly male workers worked in shelters as it is a full-time job and females were required to manage housework. As well, managing cow shelters is clearly a male-dominated profession, to add to the great imbalance in favour of male workers employed in the shelters. In a recent study on public attitudes towards cow shelters, males were more likely to credit shelters as being of religious importance [30]. Traditionally, decision making and managerial roles have been either denied or constrained for women in the animal husbandry sector in India due to the paternalistic bias of Indian society [32]. Gender inequalities favoring males exist in access to information, as well as ownership of land and livestock in Indian society [33]. The women are mostly confined to household work, including tending to livestock at home whereas the men work outside the homes to earn a stable income.

The percentage of rural and urban backgrounds of the shelter managers was almost equal to the rural and urban population in the current demography of India [34]. The majority of the managers were in the age range of 46–65 years, had graduate and postgraduate qualifications and experience of working in cattle farms, which gives confidence in their maturity, education level and experience levels to handle the complex routine management of the gaushalas. The majority of them also identified their role as being team leaders supervising the workers.

The static nature of our survey does not reflect what is a dynamic process, with intake of cattle at regular intervals into the shelters all through the year, rather than tending to a fixed number of cattle. Managers’ monthly stock records were made available in some shelters and revealed a regular influx of cattle through the year. No discrimination was observed in rearing of male and female calves in the shelters and more than half of them were reared in the shelters to adulthood. Both male and female calves (almost equal numbers of each) were donated to the villagers nearby on demand. If they were sold, it is expected that it would be for a much lower price than market value, because of the risk of them carrying disease. Over time it is likely that male calves will be in less demand due to the gradual mechanization of agricultural operations [1]; suggesting that more male animals will be abandoned. Fewer bullocks are being raised by farmers, as fodder availability and cost prohibits round their year maintenance. Renting of tractors or maintaining mechanized tillers for ploughing the fields is likely to be more economical. These factors contribute to the low demand for male calves to be raised as draft animals. Calves obtained free or at low cost are more likely to be abandoned, hence it would be better to improve disease management in the gaushalas, which would then enable the calves to be sold at market price.

### 5.2. Health Management

The majority of the cattle sheltered in gaushalas were likely to be immunocompromised, with infectious disease-causing agents like *Listeria* sp., *Streptococcus* sp., *Staphylococcus* sp., and *Corynebacterium* sp. predominating due to the unhygienic environment [35,36]. Vaccination against FMD, Black Quarter, and haemorrhagic septicaemia was satisfactory in this study. However, 4% of the shelters did not vaccinate their animals at all, which is a concern as many diseases, particularly FMD, are enzootic in India, with recurrent outbreaks leading to economic and social losses [37,38,39]. These shelters might act as potential reservoirs of the disease threatening the local cattle population. The government plays an active role through state animal husbandry departments, by distributing vaccines free of cost to the gaushalas, and in many cases offering veterinarians for the vaccinations. However, a high cost of veterinary services has been reported as one of the constraints faced by gaushalas in a couple of Indian states [29,40].

Vaccination and testing for brucellosis were rare and this could present a public health threat to the personnel working in the shelters and consumers, besides the sheltered cattle. There have been cases of Brucella positive cattle being culled by dairy farms and then sheltered in gaushalas [41,42]. A study has found a 15.5% prevalence of brucellosis in gaushala cattle and 4.5% in the workers employed in the gaushalas [43]. None of the shelters were testing their cattle for tuberculosis, a zoonotic disease with considerable public health implications. There are chances of tuberculosis positive retired cattle being admitted to the gaushalas as tuberculosis is prevalent in both the organized and unorganized dairy sector in India, generating cows for the shelters [44,45]. Gaushala cows have been found to be often positive for tuberculosis, with higher prevalence rates than organized and rural farms [46,47]. India has the world’s highest burden of human tuberculosis [48], and the possible role of gaushalas in the zoonotic transmission of this disease is a concern. Another disease of zoonotic importance, listeriosis, has been isolated from gaushala cattle; the disease is shed through faeces, vaginal secretions and can survive for prolonged time in harsh conditions, leading to increased risk of further transmission [36,49,50].

Use of both endo and ectoparasiticides was practised in most cow shelters, though the frequency of application varied widely. The prevalence of tick infestation in gaushalas and unorganized dairy farms has been reported as 45%, but only 4% in the organized sector. [51,52]. Besides the ticks feeding on blood, infestation leads to anemia and loss of body condition [53], and they transmit babesiosis, anaplasmosis and borreliosis [52,54]. Deworming in our study was more common than previously reported in a localized study [55]. A 44% prevalence of gastrointestinal parasitism has been reported in gaushalas in one part of the state of Gujarat [56], a state included in our study. Gastrointestinal parasitism and lungworms reduce growth [57,58].

The lack of permanent veterinarians in the majority of shelters is likely to hinder management of sick cows and routine health initiatives. There is no requirement for mandatory veterinary attendance at gaushalas, and a shortage of field veterinarians in India [59]. However, most of the veterinarians employed by the state animal husbandry departments provide technical assistance to the shelters located within their jurisdiction.

### 5.3. Visitors to the Shelter

Most gaushalas welcomed visitors, which suggests that they have an important social and religious function, however this will also compromise biosecurity. Moreover, many shelters reported outbreaks of FMD in the last five years, which might be due to poor biosecurity, as the majority of the shelters vaccinated their animals against the disease. FMD is endemic in India, spreading by direct contact with infected animals, fomites of workers, fodder and feeding utensils [60] with vaccination and restriction of animal movements are the core control methods [61]. Unhygienic conditions and immunocompromised animals in shelters also contribute to a high prevalence of listeriosis [36]. These highly infectious, communicable and zoonotic diseases and biosecurity and screening protocols are very important to prevent shelters being reservoirs of these diseases.

Feeding of food produced in visitors’ homes to the cows in most of the shelters might lead to gastrointestinal disturbances, e.g., ruminal acidosis or grain engorgement. There are reports of shelter cows getting sick due to eating such food in excessive quantities or eating spoilt food [62].

### 5.4. Cow Mortality

Mortality rate in cows is an indicator of health and welfare. The median mortality rate of 13.8% was higher than for dairy cows on farms in Western countries, in which it ranges between 1% and 5% [63]. There are only limited data about mortality rates of dairy cattle in India from single states, ranging from 4–20% [64,65,66]. There are no other estimates of mortality rates in shelter cattle for comparison, but it is expected that it would be higher than in dairy farms as most of the sheltered cattle are old, debilitated and infirm. This was confirmed by the shelter managers who ranked old age as the biggest cause of death. Studies on dairy cows have found the mortality to be double in old cows (≥ 6.5 years) than young cows (< 6.5 years) [63,67,68].

Post-mortems of dead animals in the shelters are advisable to identify possible causes of death but the logistics of disposal, availability of veterinarians and risk of zoonotic diseases may mitigate against them. Cows are often brought into the shelter in a moribund condition after sustaining automobile hits, being rescued from transportation to illegal slaughter houses or enduring a life of on the streets with a lack of adequate food and shelter. However, these were confirmed as less important reasons than old age as causes of mortality. Fodder shortages in overpopulated shelters may predispose cows to malnutrition, with competition for meagre fodder, such as poor quality straw. Overstocking increases aggression between the cows especially at the feed bunk, leading to injuries which may sometimes be fatal, as most of cows in shelters have horns [69,70,71]. Based on our observations during our visits to the shelters and interactions with shelter managers, the segregation of the animals on the basis of sex, age and body condition could improve their welfare.

Mortality also occurred following ingestion of plastic bags. Most of the cows had been rescued from the streets, especially in urban gaushalas they are forced to scavenge on the plastic laden garbage in bins and refuse dumps. In one study 95% of stray cattle had gastrointestinal disorders following ingestion of plastic bags and other foreign bodies [72]. Plastic ingestion causes gastrointestinal disorders such as ruminal impaction, indigestion and tympany [72,73,74] and if not treated surgically becomes fatal. Cows with plastics lodged in their stomach are also immunosuppressed, making them susceptible to other infections [72].

### 5.5. Routine Management and Waste Disposal

Most shelters were being used as rescue homes for managing the street cow overpopulation rather than breed conservation or upgradation centers. Thus, breeding of the cows in the shelters might not be desirable from an animal welfare point of view. Breeding of old and low body condition cows might compromise the health of the dam and the calf due to the lack of specific individual animal care. Moreover, further increases in animal population through such calvings could pose additional difficulties in managing the growing number of animals being admitted to the shelters. In the past shelters were encouraged as breed conservation centers by the government [75]. However, indiscriminate breeding of cows, observed in half of the shelters in our study, if not checked could severely impact the cows’ welfare due to overcrowding. Separation of calves from their mothers in 40% of the shelters is also a welfare concern. Conversely not segregating cows according to their age, body condition and length of stay in the shelters in one third of the shelters could be a reason for aggression between the cows, leading to injuries that are at times fatal.

The access to pastures in 41% of the shelters is encouraging for cow welfare; most of these shelters were located in the rural areas, whereas the cows in urban shelters did not have the benefit of pasture grazing. Pasture grazing changes the physical environment of the cows, enables them to exercise, induces changes in diet routines and improves the health of the hooves. Pasture grazing helps cows recover from lameness and allows a more comfortable surface to stand upon and lie down [76]. It facilitates behaviors such as grazing, lying and resting and reduces aggression [77]. Access to yards is also good for welfare, though it cannot replace the advantages of pasture access. The exercise, interaction and exploration of environment that cows get through outdoor access to yards also improves claw conformation [78]. Exercise improves bone and hock strength and prevents hock injuries [79], through improving circulation of blood to the limbs, enabling proper nutrition and oxygen to the horn tissues of the claws producing the horn [80]. Animal enrichment in the form of devotional music in half of the shelters in the study might help in alleviating stress. Studies on enrichment of environment of cows, especially auditory enrichment through classical and country music, have demonstrated improvement in biological functioning such as health and fitness levels to cope with stressors, reduce frustration and fulfil behavioral needs [81].

The sale, donation and vermicomposting of dung promotes organic farming, which is especially valuable in rural areas where farmers cannot afford to buy chemical fertilizers. This disposal was much less than the amount of dung generated but still useful because the land area is insufficient to absorb the quantity of dung. Mounds of excreta, bedding and fodder waste generated in the shelters are health hazards to the cows in the shelters, the workers and the public living in the vicinity. Improper management and disposal of such wastes, especially in limited spaces of urban areas, are public health and environmental risks [82], contributing to point and non-point sources of environmental pollution [83]. The offensive smell of the animal waste generated is due to the decomposition of microorganisms; releasing noxious gases such as ammonia, carbon dioxide, hydrogen sulfide, and methane that adversely impact on human health [84]. There are a number of parasites in cattle dung which can be transmitted to other cows and to humans handling it [85,86]. Cryptosporidium and giardia are two intestinal protozoan parasites with zoonotic potential that have been found in cattle in shelters and roaming in streets [86]. The dung breeding flies are potential sources of transmission of diseases and parasites in humans and animals [87,88].

Urine was used in a quarter of the shelters for processing into traditional medicine or as a biopesticide for crops. In traditional Indian medicine, cow urine is claimed to cure many chronic human health disorders [89,90,91,92,93]. It has also been used as a bio-enhancer, increasing the nitrogen content of the soil, and as a bio-pesticide through its larvicidal action on fodder crops [94,95].

### 5.6. Disaster, Human Resource and Financial Management

Disaster management plans should be present but were mostly not. As cattle sheltering increases in India new shelters might be established in areas that are uninhabited by humans such as near creeks or around forests, with their attendant flood and fire risks, in which case disaster management plans will be critical.

The availability of workers in large cow shelters has not been an issue, but small shelters sometimes encounter this problem [55]. Induction training of workers was reported in two-thirds of the shelters but is an informal training; most managers believing that workers had prior experience of working with cows when they were from rural areas. This is an area of shelter management that requires attention as managing cows in shelters is different from dairy cows, with the former being malnourished and often in poor condition when rescued from streets. They need additional and humane care as they often have a fear of humans due to previous neglect and ill-treatment on the streets. Therefore, a dedicated worker induction program is important for improving the human-animal relationship in the shelters. It should not be just skill-based training but aim at behavior modification of the workers. Research has shown that training of stock persons improves beliefs, and better behavior towards animals improves their welfare [96]. Cows are venerated by the Hindu population, hence there should be an increased emphasis on the competency levels of workers to care for the cows in shelters. Animal enrichment measures in some shelters may have helped cows to cope with stress [81] by improving biological functioning, reducing frustration, and fulfilling behavior needs. However, enrichment efforts fail if the changes effected in the cows’ environment have little practical significance to the animals, are not goal-oriented and are based on incorrect assumptions of causation of problems [97]. Environment enrichment requires finance and time, both of which are often deficient in the shelters.

The maintenance of records was variable; feed records were probably the only well-maintained records in the shelters because feed consumption involved the biggest expenditure. Maintenance of records of mortality, calving, veterinary treatment, medicines, and sales should be encouraged in all shelters. Uniformity of recording is needed in order to collect and analyze data for performance analysis, auditing, interventions by advisory services and for future planning. Volunteering (regular or occasional) by the public, at least in half of the shelters, shows the connection of the people to the shelters, either due to veneration or simply for animal welfare. The outreach activities organized in the majority of the shelters focused on religious festivals ascribed to the ‘holy cow’, which could promote more volunteering. Teaching the religious scriptures on the holiness of the cows in ancient texts narrating the works of the saints might influence the spirituality of the attendees. However, a more proactive approach to shelter management with advertisements for volunteers will further enhance participation of the local public in shelter management.

The ancient nature and connection of most of the shelters with the three main religions in India (Hinduism, Buddhism, and Jainism) proves the religion-driven concept of sheltering cows. The reliance of shelters on private funding or charitable societies or trusts confirms the findings of Bijla and Singh [98]. Almost all shelters audited their funds annually, reflecting their accountability to the donors. This could be why less than half were affiliated with the AWBI, as they were not financially dependent on AWBI to function. However, AWBI is a statutory government body established as a watchdog of animal welfare all over the country and has affiliated shelters. Implementation of this as a mandatory requirement will be important to bring about uniformity in the management of cow shelters up to modern scientific standards of animal welfare, which should be determined by welfare auditing.

Most of the shelters reported higher expenditures than incomes but some were reluctant to share the exact figures of the finances. Feeding incurred the highest expenditure, which corroborates the findings on the only economic study of cow shelters, in one state of India [98]. Positive returns were reported by these researchers as the shelters were able to meet their operating costs in their study, in contrast to our study, though the median annual income by shelters in our study was approximately similar to the cited study. The reason for this could be the active support of the government of that particular state to support self-sustainability in its cow shelters, through the sale of milk and other products. The shelters studied in this study were mostly functioning as rescue homes without any economic returns, a function of our selection criteria. Adequate welfare levels in the shelters were asserted by most managers, though intentions to further improve welfare and their knowledge levels were evident. Animal welfare mostly meant care of the holy cow for them, as revealed by the qualitative analysis of the open-ended question posed to them. Financial support from the local community was acknowledged but financial support from the government was not, despite free fodder, vaccination and veterinary support being provided by the government. Shelter managements need to be made aware of the financial costs of these free services being provided by the government.

### 5.7. Associations between Shelter Administration, Affiliation, Income with Health and Welfare of Cows

The shelters affiliated to the AWBI revealed of trend of garnering more income. It could the existence of a proper managemental structure in such shelters that might encourage the public to donate money. Moreover, AWBI also provides financial and material assistance to its affiliated shelters regularly. The positive association of mortality rate with milk yield might be due to more attention of the shelter management to the dairy cows for milk production and sale than the non-productive ones, leading to the neglect and deteriorated health of the latter. High mortality rate in shelters that had in house veterinarian could be due to the high admission of cattle into such shelters. A high intake might force the shelters to hire a permanent veterinarian to cater for the upkeep of the health of the larger cattle numbers rescued from streets and slaughter. Similarly, shelters run by public trusts had significantly better biosecurity measures for newly admitted cattle than those run by charitable societies. This might be due to shelters in the public domain being more open to public scrutiny and accountable. The shelters that did not acknowledge financial support of the government were more likely to more frequently vaccinate their cattle than those that agreed that the government financially supports them. This relationship could be misleading because the government invariably provides free vaccines to all shelters in order to prevent the spread of diseases from shelter animals to the farmer owned animals. A possible explanation could be that such shelters might be financially sound and hence more efficient in safeguarding the health of their cattle.

### 5.8. Attitudes of Shelter Managers

All the shelter managers had a high opinion of the adequacy of the welfare of cows, their own work and the human-animal relationships in their respective shelters. However, almost all of them were open to adopt measures to improve the welfare of cows under them and believed that they had made improvements towards cows in their shelters. Animal welfare and public livelihood are interconnected in India [99] and the role of managers in cow shelters is one of such manifestations. The majority of the managers were Hindus from rural backgrounds, having grown up around cows with respect and reverence for cows in their religious beliefs. This could be the reason for many believing themselves to be knowledgeable and taking good care of the welfare of cows in shelters. Animals such as the cow which humans perceive as attractive are shown more empathy [100,101]. However, scientifically supported and prescribed guidelines for cow welfare might not be known to the managers. There is a willingness of stakeholders to improve animal welfare, based on science in India [99]. Most shelter managers acknowledged the moral, and to a lesser extent financial, support provided by the public. However, even though moral support by government was generally acknowledged, their financial support was acknowledged by only half of the shelter managers. In this study, government provided most of the fodder (straw) and vaccination against endemic diseases. This might not be construed as financial support by managers but can offset a major part of the running costs of the shelters. Similarly, volunteering by local people can offset labor costs.

The analysis of the qualitative assessment indicated that, despite earning their livelihood through the management of the cow shelters, the managers held cows in high esteem—as a mother goddess which must be properly cared for and should be rescued from abandonment as a part of their religious duty. The qualitative analysis of the open-ended question defined the status of the cow as being ‘holy’ and ‘mother goddess’ and the concern of the managers for its abandonment and its proper care after rescue from slaughter. This result to some extent reveals the exalted status of the cow among the Hindus in contemporary India and the understanding about its welfare.

## 6. Limitations of the Study

The selection of the shelters based on the suggestions from the AWBI, veterinarians from the states covered in the study and snow-balling might generate selection bias as shelters with different levels of welfare and size were studied.

There is a possibility that managers might have tried to report to the researcher answers that the researcher wanted. However, the face-to-face interview technique has less chance of false reporting than other techniques of data collection. It is also possible that 54 shelters in six states were not representative of all the shelters in India, but logistical, time and financial constraints made us select a statistically viable sample to report the contemporary situation of shelters in just the states which had a tradition of sheltering, and one state, Himachal Pradesh, where there was a government initiative to open new cow shelters.

This research is the first survey of contemporary cow shelter management through a cross-sectional study, which has its inherent limitations and biases. More studies are required to find out the regional differences, issues and constraints in the management of cow shelters in all states of India. Longitudinal studies should also be undertaken to observe the effects of government interventions on the strengthening as well as opening of shelters. Economic analysis of the sheltering of cows also needs more in-depth and focused studies.

## 7. Conclusions

Managers are very important stakeholders in the welfare of cows in shelters. They are in an ideal position due to their work profile and experience to identify the problems and constraints of the routine management of shelters. Therefore, their engagement in all initiatives to improve welfare of cows in shelters is vital for the perpetuation of the sustainability of these unique traditional institutions. Sheltering of cows is a dynamic process, with abandoned and rescued cows regularly entering the shelters. Biosecurity measures in the shelters do need enhancing, to prevent shelters becoming reservoirs of infections, parasitism, and zoonotic diseases. Specific shelter protocols need to be formulated at a national level and enforcement of compliance of these protocols ensured through a central governing body. A greater involvement of qualified veterinarians would benefit the management of animal health. There was evidence from this study of involvement of permanent veterinarians in shelters that witnessed high mortality rates. This also suggests increasing cow numbers in shelters in future would invariably need in house veterinarians to cater to the health needs of the sheltered cows, but that this might not necessarily prevent an increase in mortality rate.

This study identified various welfare issues through the survey of shelter managers that can be resolved by managemental initiative and intervention. Indiscriminate breeding, lack of access to pasture and tethering of cows are the welfare issues that demand a comprehensive policy regulation encompassing all shelters in the country. Proper and complete disposal of dung and urine needs attention as due to increasing cow numbers as well as shelters, this poses a public health risk. Feeding of cows by visitors needs routine monitoring. A uniformity in the maintenance of all records in all shelters throughout the country is important. This will help in welfare interventions, support, auditing and feedback for all stakeholders. Mandatory affiliation of all shelters to the AWBI is desirable, given its statutory role as an advisor and watchdog of animal welfare in the country. Evidence of shelters affiliated to AWBI being able to generate more income in this study also justifies the above recommendation. Affiliation to AWBI is mandatory to receive regular funding from the AWBI and helps in convincing the donors (especially the general public) about the proper utilization and accountability of their donations. Cow shelters can become educational centers for animal welfare through their outreach programs. Shelters run by public trusts were more vigilant towards biosecurity measures than those run by charitable societies. This suggests value in further strengthening of public trusts in cow shelter management, and that ensuring better compliance to biosecurity protocols in shelters runs by charitable societies would be a worthwhile aim. Shelter management need to understand that vaccines entail a huge financial cost to the government, as they are provided free of cost to the shelters along with logistic support by government veterinarians and support staff. This, if accounted into financial terms is a strong support from government to the shelters, which is unfortunately not recognized by many shelter managers. The issue of euthanizing very sick cows and those suffering with contagious diseases needs careful deliberation with the stakeholders, taking into account the strong religious reservations to it. Animal welfare scientists, policy makers and public representatives need to deliberate on the humaneness of euthanasia in such exceptional cases.

Welfare centric training of managers and workers should be easily implemented, as all the managers were willing to accept suggestions to improve the welfare of cows in their respective shelters. Training of managers and workers in modern scientific concepts of welfare-based management of cattle will help in an excellent amalgamation of science and tradition to sustain this institution of sheltering cows, which signifies the perpetuation of some traditional ethos of Indian society towards cow welfare.

## Figures and Tables

**Table 1:** Word frequency count of the question ‘What do you understand by the term ‘welfare of cows’?

Word	Length	Count	Weighted Percentage (%)	Similar Words
care	4	27	16.56	care, cared, cares
mother	6	16	9.82	mother
goddess	7	16	9.82	goddess
rescued	7	12	7.36	rescue, rescued
abandoned	9	10	6.13	abandoned, abandonment
feeding	7	9	5.52	feeding
proper	6	8	4.91	proper
duty	4	4	2.45	duty
freedom	7	4	2.45	freedom, freedoms
religious	9	4	2.45	religious
watering	8	4	2.45	watering
dumb	4	3	1.84	dumb
heritage	8	3	1.84	heritage
protected	9	3	1.84	protected, protection, protections
slaughter	9	3	1.84	slaughter
five	4	2	1.23	five
granting	8	2	1.23	granting
service	7	2	1.23	service

## Supplementary Materials

The following are available online at https://www.mdpi.com/2076-2615/10/2/211/s1.

## Appendix A

**Survey Questionnaire of the Managers on Welfare of Cattle in Shelters (Gaushalas) in India.**

**Gaushala Name & Code:**

**Screening questions**

1. Does this gaushala have a minimum of 30 animals?
  - Yes
  - No
2. Which of the following are admitted to this goshala?
  a) Infertile
  b) abandoned
  c) infirm
  d) old cows
  e) Stray cows
  f) All of these
  g) Other
3. What religious connection does it have?
  a) Jain
  b) Hindu
  c) Sikh
  d) Other
4. When was this gaushala established? _________________________________

**PART 1- Demographics**

1. Please indicate your gender
  - Male……….... □
  - Female…….... □
2. In what type of area have you lived for most of your life?
  - Urban (city)…................................ □
  - Sub – Urban (Suburb)……........... □
  - Country town (Tehsil/Taluka) .... □
  - Village…………………………...... □
3. Please indicate your age range
  - 18–25……............ □
  - 26–35……............ □
  - 36–45…….......…. □
  - 46–55…….......…. □
  - 56–65…….......…. □
  - Over 65……........ □
4. Please indicate your education level
  - No formal education………….... □
  - Below 10^th^ class…….................... □
  - 10^th^ class (Higher secondary)…. □
  - 10 +2 (senior secondary)….……. □
  - Diplomate
  - Graduand…….......................…. □
  - Post-graduand……...............…. □
  - Other, if any.............................. □
5. Which religion do you follow?
  - Bahai’ Faith…….......... □
  - Buddhism……............. □
  - Caodaism……............. □
  - Chinese folk religion.. □
  - Chondogyo………...... □
  - Christianity……......... □
  - Confuciansim……...... □
  - Hinduism……............. □
  - Islam……..................... □
  - Jainism……................. □
  - Judaism……................ □
  - Shinto……................... □
  - Sikhism……................ □
  - Taoism……................. □
  - I don’t follow a religion□
  - Atheism........................□
  - Other (please specify) _______________________________________
6. To what extent do you consider yourself religious?
  - Not religious at all…...... □
  - Not very religious.......... □
  - Moderately religious..... □
  - Very religious…….......... □
7. Please indicate which job role best describes your involvement in the gaushala
  - Work directly with the animals…………………………………………… □
  - Team Leader: Supervise people who work directly with the animals.. □
  - Business owner…………………………………………………………….... □
  - Business Manager…………………………………………………………... □
  - Farmer………………………………………………………………………... □
  - Veterinarian who treats animals’ hands on……………………………… □
  - Veterinarian working for the Government as an advisor……………… □
  - Other, if any…………………………………………………………………. □
8. Please indicate your level of understanding of gaushalas
  - Expert ……………………………………………………………………......... □
  - Good knowledge……………………………………………………………... □
  - Some knowledge…………………………………………………………….... □
  - Little knowledge……………………………………………………………… □
  - No knowledge………………………………………………………………… □
9. How did you gain your knowledge about cow welfare in goshalas?
  - Formal qualifications – relevant degree, training course……….…….......................... □
  - Farm employment – hands on experience………………………………........................ □
  - Personal interest – internet, journals, newspaper articles, television programmes... □
  - Friends and acquaintances……………………………………………….......................... □
  - Other ……….…………………………………………………………….…........................ □
10. Please indicate the type of animal welfare organisation you have been involved with other than this gaushala
  - Gaushala…………………………………………. □
  - Dairy industry ………………………………….. □
  - Animal Welfare organisation………………….. □
  - Other…………………………………………….... □
  - None……………………………………………… □
11. Please indicate the type of animal welfare activity you have been involved with in addition to managing this gaushala
  - Activism……………………….………………… □
  - Advocacy …………………………….………..... □
  - Administration ……………………………........ □
  - Policy making…………………………………… □
  - Feeding street/stray animals…………………… □
  - Humane Education……………………………... □
  - None………………………………………………. □
12. Please indicate how long you have been working in the field of animal welfare
  - Less than 1 year…………………………………………………………….□
  - 2 – 3 Years…………………………………………………………………. □
  - 3 – 5 Years…………………………………………………………………. □
  - 5 – 9 Years…………………………………………………………………. □
  - 10 – 15 Years……………………………………………………………… □
  - More than 15 Years………………………………………………………. □
13. Please indicate how long you have been working in this gaushala
  a) Less than 1 year………………………………………………………….... □
  b) 2 – 3 Years…………………………………………………………………. □
  c) 3 – 5 Years………………………………………………………………….. □
  d) 5 – 9 Years…………………………………………………………………. □
  e) 10 – 15 Years……………………………………………………………….. □
  f) More than 15 Years………………………………………………………... □

**PART 2 – Complementary Data**

1. No. of cattle entering the gaushala
  a) In last 3 months  __________________
  b) In last 6 months  __________________
  c) In last 1 year  __________________
  d) No records kept  __________________
2. Total milk yield of the gaushala/ day
  a) ______________ litres/day
  b) No records kept
3. No. of lactating cows in the gaushala
  a) ___________ cows
  b) No records kept
4. Approximate proportion of horned animals
  __________%
5. No. of males and female animals in the gaushala
  a) Bulls
  b) Cows
  c) Heifers
  d) Bullocks
  e) Male calves (6 month or less)
  f) Female calves (6 month or less)
6. If calves are born in the gaushala, what do you do with the calves?
  a) Male calves
    i) Sell
    ii) Donate
    iii) Rear
  b) Female calves
    i) Sell
    ii) Donate
    iii) Rear
7. Vaccination status of the animals
  a) Vaccinated
  b) Non-vaccinated
  c) Some vaccinated, some not
8. If vaccinated, vaccinated against which diseases –
9. If vaccinated how many times vaccination done
  a) one a year
  b) twice a year
  c) thrice a year
  d) four times a year
  e) No regular schedule followed
  f) Never done
10. Deworming status of the animals
  a) Dewormed
  b) Non-dewormed
  c) Some dewormed, some not
11. If dewormed how many times deworming done
  a) one a year
  b) twice a year
  c) thrice a year
  d) four times a year
  e) No regular schedule followed
  f) Never done
12. If ectoparasiticidal treatment given?
  a) one a year
  b) twice a year
  c) thrice a year
  d) four times a year
  e) No regular schedule followed
  f) Never done
13. Veterinarian in the gaushala: In house / Visiting (If visiting how frequent)
  a) Daily
  b) Weekly
  c) Fortnightly
  d) Monthly
  e) On call
14. No. of workers in the gaushala: Male _____________ Female ______________
15. Training of animal workers is done
  a) Induction training done
  b) Not done at all
  c) Trained workers inducted
16. Maintenance of records in the gaushala: List of records
  i) Milk yield
  ii) Calving/Reproduction
  iii) Health Records
  iv) Veterinary provisions/inventory
  v) Mortality
  vi) Feeding
  vii) Sales
17. Sale of livestock products
  a) Milk: Yes / No
  b) Dung: Yes / No
  c) Urine: Yes / No
  d) Carcass: Yes/No
18. Do you have a biogas production system?
  a) Yes
  b) No
19. Who runs the administration of the gaushala?
  ______________________________
20. Rank any of the following which are funding sources, in declining order of importance
  a) State Government           □
  b) Central Government         □
  c) Both the central and state government  □
  d) Trust                □
  e) Philanthropy             □
  f) Temple Trust             □
  g) Foreign Funding          □
  h) Others, if any            □
21. Is the gaushala affiliated to AWBI?
  a) Yes
  b) No
22. Mortality rate in the gaushala ___________deaths/year.
23. Rank any of the following which are the causes of death?
  a) Old age           □
  b) Debility          □
  c) Malnutrition        □
  d) Disease           □
  e) Brought in moribund state  □
  f) Others           □
24. What does ‘cow welfare’ mean to you?
  Key words
  --------------------------------------------------------------------------------------------------------------------------------
25. Do you feed colostrum to the calves? Yes / No. If Yes, then
  i) To male calves
  ii) To female calves
  iii) To both
26. When do you feed colostrum to the calves?
  i) Immediately after birth
  ii) After 6 hours of birth
  iii) After 12 hours
  iv) After 24 hours
  v) After 48 hours
27. Do you separate the calf from the mother after birth?
  i) Yes
  ii) No
28. What is the feeding regime of your gaushala? (Schedule and formulation)
29. How do you manage the male calves born in the gaushala?
  i) Maintain them in the gaushala for rearing as breeding bulls
  ii) Sell them
  iii) Donate them
  iv) Castrate other than those kept for breeding
30. How much time is spent by the animals outdoors in the yard or in the grazing land in a day?
  i) Not sent out at all
  ii) 1–2 hours
  iii) 2–4 hours
  iv) 4–6 hours
  v) More than 6 hours
31. Is breeding of cows done in the gaushala?
  i) Yes
  ii) No
32. If yes to Q.32, then what type of breeding?
  i) Indiscriminate
  ii) Natural breeding from a bull present in gaushala
  iii) Artificial insemination
33. What is the purpose this breeding?
  i) Breed improvement/improvement
  ii) Improve productivity
  iii) No purpose
34. Are the funds received by the gaushala audited regularly?
  i) Yes, always
  ii) Sometimes
  iii) No
35. How long the workers are working in the gaushala?
  i) 6 months
  ii) 1 year
  iii) 2 years
  iv) 3 years
  v) More than 3 years
  vi) Keep on leaving frequently
36. 36. How long you are working in the gaushala (manager)?
  i) Less than 1 year
  ii) 1–2 years
  iii) 3–5 years
  iv) More than 5 years
37. Do people come for volunteering in the gaushala?
  i) Yes, regularly
  ii) No
  iii) Occasionally
38. What type of voluntary work is done?
  -------------------------------------------------------------------------------------------------------
39. Are there any animal enrichment measures in place in the gaushala?
40. Are there any biosecurity measures in place in the gaushala?
  Introduction of new animals
  Disposal of carcasses
  Isolation room for animals suffering from infectious diseases
41. Was there any disease outbreak in 5 years in the gaushala? If yes, what was it?
42. Is there any hierarchy of animals in the animal groups and how is it controlled?
43. Is there any public relation or outreach activity done by the gaushala involving the local community?
44. Is the gaushala located in a drought prone or flood prone area/ disaster prone area?
  i) Yes
  ii) No
45. Are there any disaster preparedness plans in place?
46. Are the records of visitors maintained?
47. What is the purpose of visit of the visitors?
48. Is the feeding of animals by the visitors monitored by the management?
49. How is the Disposal of dung carried out?
50. How is the Handling/disposal of urine done?
51. Are dung/urine utilized as value-added resources?
52. Are there loading and unloading ramps for cows in the gaushala?
53. Is animal experimentation allowed in the gaushala?

**Part -3 Attitudes**

	**Strongly Disagree**	**Disagree**	**Neither Disagree or Agree**	**Agree**	**Strongly Agree**
**1.** The welfare of the cattle in this gaushala is satisfactory.					
**2.** The welfare of the cattle in the gaushala is important to me.					
**3.** I feel that my knowledge of animal welfare is adequate.					
**4.** The feed the cattle receive at this gaushala is adequate.					
**5.** I am willing to adopt measures that will improve the welfare of the cattle, if it was provided to me.					
**6.** The local community financially supports this gaushala.					
**7.** The local community morally supports this gaushala.					
**8.** The government financially supports this gaushala.					
**9.** The government morally supports this gaushala.					
**10.** I intend to make improvements to the welfare of the cattle in my care.					
**11.** In the past I have tried to make improvements to the welfare of the animals in my care.					
**12.** The staff at this gaushala have a close relationship with the cattle.					

## Appendix B

**Table A1:** Mean responses to various attitudes questions posed to cow shelter managers on a scale of 1 strongly disagree to 5 strongly agree (r^2^ = 31.4%).

Factor	Mean	SEM	95% CI
The welfare of the cattle in the gaushala is important to me.	4.92 ^a^	0.035	4.70–5.14
I am willing to adopt measures that will improve the welfare of the cattle if it is provided to me.	4.83 ^ab^	0.063	4.61–5.05
The feed the cattle receive at this gaushala is adequate.	4.81 ^ab^	0.075	4.59–5.03
In the past, I have tried to make improvements to the welfare of the animals in my care.	4.79 ^ab^	0.055	4.57–5.01
The staff at this gaushala have a close relationship with the cattle.	4.79 ^ab^	0.071	4.57–5.01
I intend to make improvements to the welfare of the cattle under my care	4.75 ^ab^	0.074	4.53–4.97
The welfare of the cattle in this gaushala is satisfactory.	4.57 ^abc^	0.117	4.35–4.79
I feel that my knowledge of animal welfare is adequate.	4.35 ^bc^	0.109	4.13–4.57
The local community morally supports the gaushala.	4.18 ^cd^	0.152	3.96–4.40
The government morally supports the gaushala.	3.72 ^d^	0.133	3.50–3.94
The local community financially supports the gaushala.	3.70 ^d^	0.184	3.48–3.92
The government financially supports the gaushala.	3.07 ^e^	0.158	2.85–3.29

Means with different superscript differ significantly (*p* < 0.05) by Tukey’s test.
